# Fluoroscopic-Guided Removal of Jejunal Sharp Foreign Body: An Alternative Approach to Surgery

**DOI:** 10.1155/2024/5117360

**Published:** 2024-09-16

**Authors:** Abdulrahman Qatomah, Simon McQueen, Wafa Qatomah, Aishah Qatomah, Ali Bessissow

**Affiliations:** ^1^ Department of Gastroenterology and Hepatology McGill University, Montreal, QC, Canada; ^2^ Division of Gastroenterology and Hepatology King Faisal Specialist Hospital and Research Center, Jeddah, Saudi Arabia; ^3^ Department of Radiology McGill University, Montreal, QC, Canada; ^4^ Department of Radiology King Abdulaziz Medical City, Riyadh, Saudi Arabia; ^5^ Department of Medicine Assir Central Hospital, Abha, Saudi Arabia

## Abstract

**Introduction:**

Foreign body (FB) ingestion represents a frequently encountered scenario in clinical practice. Most ingested FBs typically pass spontaneously, requiring no intervention. Endoscopic removal stands out as the least invasive method, with only a minimal 1% needing surgical intervention. *Case Presentation*. We present a case of a 30-year-old male who ingested multiple FBs located in the stomach and small bowel. While successful removal of the stomach FB was achieved through endoscopy, the second FB in the small bowel proved challenging due to perforation concerns and limited expertise. Given a history of prior surgical intervention resulting in a large incisional hernia, surgical removal was discouraged. Consequently, a collaborative decision involving surgeon and interventional radiologist (IR) led to the adoption of a fluoroscopic-guided removal approach facilitated by IR techniques.

**Conclusion:**

This case highlights the potential for a less invasive alternative in situations where both endoscopic and surgical interventions are deemed not feasible.

## 1. Introduction

Foreign body (FB) ingestion is more frequently encountered in the pediatric population [1]; however, besides children, FB ingestion usually happens to psychiatric, incarcerated, and elderly frail patients. Most FBs (80–90%) will eventually pass spontaneously without intervention, and only 1% will require surgical extraction [2]. It is recommended to remove sharp-pointed FB that fail to pass through the gastrointestinal tract within 24 hours. This presents challenges to gastroenterologists since these FBs can be hard to retrieve endoscopically and carry a high rate of mucosal injury [3–6]. Herein, we report a case of a sharp-pointed FB in the proximal small bowel that was removed via fluoroscopic guidance by interventional radiology.

## 2. Case Presentation

A 30-year-old male with a history of borderline personality disorder and bipolar disorder presented to our emergency department with a 3-day history of abdominal pain following the ingestion of 2 sharp-pointed FBs. The patient disclosed ingesting a hair removal forcep (“tweezer”) and a small sewing needle. He had a prior history of FB ingestion, one of which required surgical removal via laparotomy complicated by an incisional hernia.

**Figure 1:**
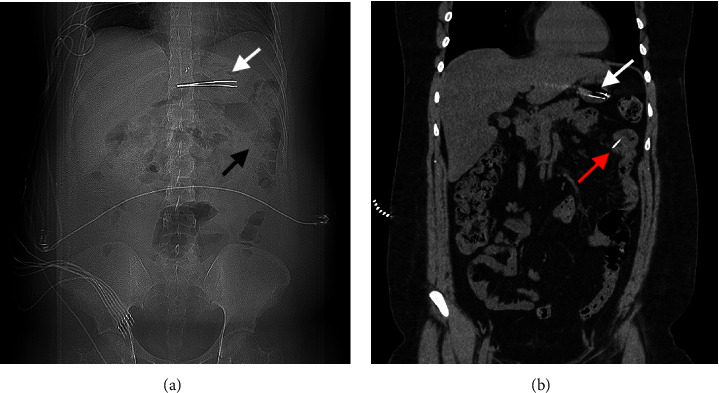
(a, b) Scout CT image showing both FB in the stomach (white arrow) and the small bowel (black arrow), respectively. Coronal CT scan showing both objects in the stomach (white arrow) and the small bowel (red arrow).

Physical examination revealed a long midline scar with a reducible incisional hernia upon mild palpation and mild generalized abdominal tenderness without signs of peritonitis. Laboratory work ups, including complete blood counts, renal profile, C-reactive protein, and venous blood gas, were all unremarkable. The patient underwent a chest X-ray which, aside from surgical clips in the epigastric area, did not reveal any foreign body. A computed tomography (CT) scan showed a large linear radiodense foreign body located within the gastric antrum without any signs of gastric perforation and a second smaller linear density located in the proximal jejunum, partly protruding within the jejunal mesentery with no associated extraluminal gas or inflammatory changes Figures 1(a) and 1(b).

**Figure 2:**
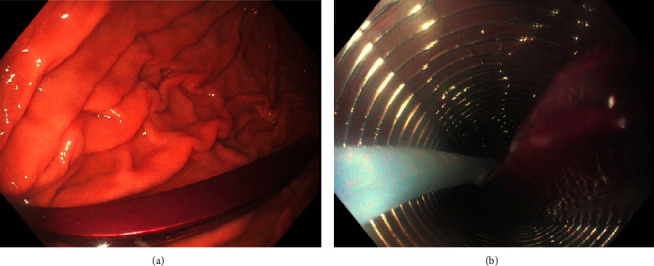
(a, b) Endoscopic images of the foreign body (tweezer) removed from the stomach.

Gastroenterologist and general surgeon consultations were requested. The patient underwent successful endoscopic removal of the FB from the stomach using an overtube, after intubating the patient (Figures 2(a) and 2(b)). The gastroscope was then advanced further into the proximal jejunum to its maximum length but failed to reach the second FB. We explored the possibility of endoscopic removal using balloon enteroscopy; however, this was later discouraged due to safety concerns about potential perforation exacerbated by CO_2_ insufflation. Considering the patient's age and prior laparotomy with a medium-sized incisional hernia, the surgical team opted for a nonsurgical approach. Liaise with interventional radiology led to the decision to proceed with fluoroscopic-guided removal of the FB via interventional radiology.

## 3. Procedure

**Figure 3:**
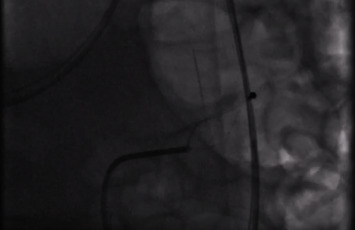
Trapezoid basket attaching to the proximal blunted end of the sharp-pointed object.

The patient was intubated during the procedure. After the initial fluoroscopy image, a 5 French (Fr) Kumpe (KMP) catheter was used for orogastric cannulation to the stomach. A 0.035 Terumo hydrophilic guidewire was then advanced successfully to the duodenum, passing the ligament of Treitz, which allowed further advancement of the KMP catheter into the small bowel. The wire was then exchanged with a Boston Scientific 260-millimeter (mm) exchange length Amplatz wire to increase the system stability. The KMP catheter was exchanged for a 10Fr Arrow metallic sheath thereafter. An initial attempt to advance both the sheath and catheter into the jejunum was unsuccessful, and the catheter was pulled back into the stomach. We then elected to use a 32 mm Coda balloon to provide counter-traction, aiming to straighten the jejunal loop with partial success. The guidewire, however, was able to reach the level of the foreign body as confirmed by live fluoroscopy. With careful maneuvering of both the sheath and the guidewire using forward and backward movements, the sheath was advanced to the level of the FB. A Boston Scientific Trapezoid Rx 3-centimeter wire-guided retrieval basket was advanced through the sheath with a straightforward exit and was able to capture the proximal blunt end of the needle, allowing for safer extraction of the FB by trailing the sharp end with little chance of tethering or lacerating the intestinal mucosa on withdrawal (Figure 3). The needle was removed successfully, and the final fluoroscopic image at the end of the procedure showed no free air in the peritoneum.

## 4. Discussion

Sharp-pointed FB removal represents a challenge for both endoscopists and surgeons. Many factors play an important role in deciding the appropriate pathway, including the patient's age, the site of the FB impaction, and the presence of FB-related complications. The goal is to safely remove the FB using the least invasive method. The use of fluoroscopy to guide endoscopic removal of FBs has been described in the literature. Removal of FB in our case exposed several challenges, including the lodgment of the FB into the jejunal lumen, limited access to an experienced endoscopist who could have performed balloon enteroscopy, and the patient's history of prior surgery with a medium-sized incisional hernia. Utilizing the expertise of a skilled interventional radiologist was key to the success of our case, resulting in a short hospital stay of 1 day with no adverse events during or following the procedure. This opens a window for IR as a salvage approach to retrieve FBs under fluoroscopic guidance. Although there is a theoretical risk of laceration of the mucosa or perforation, this remains a minimal concern.

## 5. Conclusion

Removal of sharp-pointed objects from difficult locations in the gastrointestinal tract can be achieved using a specialized IR technique. More studies are needed to highlight the feasibility and outcomes of such an approach.

## Data Availability

All data generated or analyzed during this study are included within the article and are also available from the corresponding author upon request.
